# Mechanisms of Ligand Hyperfine Coupling in Transition-Metal Complexes: σ and π Transmission Pathways

**DOI:** 10.1021/acs.inorgchem.3c04425

**Published:** 2024-05-01

**Authors:** Jan Novotný, Markéta Munzarová, Radek Marek

**Affiliations:** † CEITEC − Central European Institute of Technology, Masaryk University, Kamenice 5, Brno CZ-62500, Czechia; ‡ Department of Chemistry, Faculty of Science, Masaryk University, Kamenice 5, Brno CZ-62500, Czechia

## Abstract

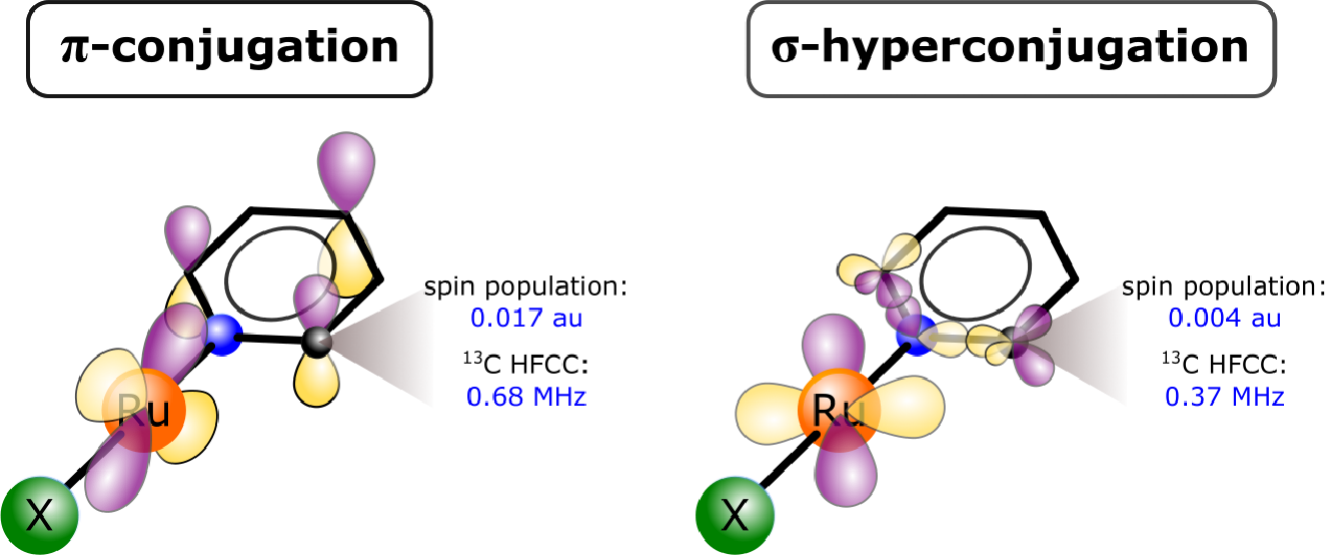

Theoretical interpretation of hyperfine interactions was pioneered in the 1950s–1960s by the seminal works of McConnell, Karplus, and others for organic radicals and by Watson and Freeman for transition-metal (TM) complexes. In this work, we investigate a series of octahedral Ru(III) complexes with aromatic ligands to understand the mechanism of transmission of the spin density from the d-orbital of the metal to the s-orbitals of the ligand atoms. Spin densities and spin populations underlying ligand hyperfine couplings are analyzed in terms of π-conjugative or σ-hyperconjugative delocalization vs spin polarization based on symmetry considerations and restricted open-shell vs unrestricted wave function analysis. The transmission of spin density is shown to be most efficient in the case of symmetry-allowed π-conjugative delocalization, but when the π-conjugation is partially or fully symmetry-forbidden, it can be surpassed by σ-hyperconjugative delocalization. Despite a lower spin population of the ligand in σ-hyperconjugative transmission, the hyperfine couplings can be larger because of the direct involvement of the ligand s-orbitals in this delocalization pathway. We demonstrate a quantitative correlation between the hyperfine couplings of aromatic ligand atoms and the characteristics of the metal–ligand bond modulated by the *trans* substituent, a hyperfine *trans effect*.

## Abstract 2

The spin transmission underlying the Fermi-contact mechanism of the isotropic hyperfine coupling is elucidated through the concepts of π-conjugation and σ-hyperconjugation delocalization, as well as by neighbor-atom and resonance polarization. The influence of ligand X on the pyridine hyperfine coupling is demonstrated and interpreted employing the hyperfine *trans* effect.

## 1 Introduction

Electron paramagnetic resonance (EPR) spectroscopy has traditionally been used to investigate systems containing unpaired electron(s).^1−7^ The initial EPR studies involved small organic radicals and biradicals, small inorganic radicals, and open-shell transition metal (TM) ions. Later, EPR spectroscopy matured into a very important technique in biochemistry for analyzing radicals in low-symmetry protein environments with specific hydrogen bonding interactions, mono- and polynuclear metal active sites in metalloproteins, and short-lived charge-separated states in photosynthesis.^8−11^ It is also used in materials chemistry to monitor metals or radicals in zeolites, metal–organic frameworks, and other prospective materials.^12,13^

The fundamental EPR information about the paramagnetic system is obtained from the electronic **g**-tensor, the hyperfine coupling (HFC) tensor (**A**), and, in the presence of more than one unpaired electron, also the zero-field splitting tensor.^14,15^ For TM radicals, the distribution of the spin density at the paramagnetic center can be deduced from the metal HFC tensor, and its transmission to other parts of the system can be investigated from the ligand HFC tensor.^16,17^ Therefore, the ligand HFC constant (HFCC) is an excellent indicator of the nature of the metal–ligand bond, including its covalency. The ligand HFCC has relatively rarely been measured by EPR and reported for atoms more than two chemical bonds from the paramagnetic metal center. However, information about the long-range hyperfine interaction (more than two chemical bonds) can alternatively be extracted from paramagnetic NMR spectroscopy as a ligand hyperfine NMR shift.^18,19^

At the nonrelativistic level of theory, the ligand HFC tensor is composed of the Fermi contact (FC) and spin-dipolar terms. The FC part results from the direct interaction of the nucleus with the electron-spin density and is proportional to the spin density at the magnetic nucleus of the ligand. In contrast, the spin-dipolar mechanism is based on the through-space interaction of the nuclear and electron spins and is proportional to the anisotropy of the spin density around the magnetic nucleus.^20,21^ The picture changes at the spin–orbit relativistic level via an additional paramagnetic spin–orbit mechanism. This mechanism can dominate the hyperfine interaction of ligand atoms directly bonded to a paramagnetic metal center,^22−24^ it can contribute significantly to the NMR shifts of some ligand atoms,^25^ and it is important at the NMR long-distance limit.^20,26,27^

Nevertheless, it has been extensively demonstrated that the HFCs of ligand atoms in TM complexes are typically dominated by the FC contribution. The spread-out of the spin density from the TM to the ligand can be seen as parallel to the transfer of spin-density from a π-electron system to hydrogen or methyl groups in neutral organic radicals. The notion of “spin polarization” was first employed by McConnel in 1956 in the context of ^1^H hyperfine interactions in aromatic free radicals. In 1961, Karplus and Fraenkel used a variation-perturbation approach to develop the famous Karplus–Fraenkel equation for HFCs of ^13^C, considering the π-electron density at the carbon in question and its nearest neighbors.^28^ The topic was expanded in a 1966 work by Lazdins and Karplus^29^ who employed a semiempirical configuration interaction to achieve separation of the “electron transfer” and “exchange polarization” contributions for allyl and ethyl radicals. The same problem was considered by Colpa and de Boer, who interpreted the ^1^H HFCs of the CH_2_ group in paramagnetic aromatic systems using the concepts of “hyperconjugation” vs “spin polarization.”^30,31^ The concepts of “exchange polarization” and “spin polarization” were shown to be equivalent for the special case in question.

For TM complexes, the mechanism of ligand HFC was first considered by Watson and Freeman in 1963.^32^ The authors employed an RHF vs UHF approach for iron-series fluorides to account for “the spin (or exchange) polarization of the F^–^ electrons by the net spin density of the unfilled 3d shells on the cation.” Further work on TM complexes could be done only in the 1990s with the availability of density-functional approaches that enabled the inclusion of electron correlation effects at an acceptable computational cost. In 1998, Cano, Ruiz, Alvarez, and Verdaguer^16^ explored a series of octahedral, square pyramidal, and binuclear complexes of V^2+^, Cr^3+^, Mo^2+^, W^2+^, Mn^2+^, Ni^2+^, and Cu^2+^. For a model complex of [CrCl_6_]^3–^, the authors noticed that “spin polarization” was much more sizable for MOs composed of TM 3d orbitals than for those formed by metal 4s and 4p AOs. In 2020, Munzarová et al.^14^ reported a detailed study of the mechanisms of EPR hyperfine coupling in TM complexes which emphasized the transfer of spin density from the metal d-type valence MOs to metal core–shell s-type AOs to understand the TM hyperfine coupling. In agreement with the work of Cano et al.,^16^ metal core–shell spin polarization was found proportional to the metal d-orbital spin population and almost independent of the metal s- and p-type spin populations.

Our current analysis of spin-polarization effects consists in comparing restricted open-shell vs unrestricted spin density distributions, orbital compositions, and orbital energies. It is well known that the inclusion of spin polarization via the unrestricted Kohn–Sham formalism can lead to significant spin contamination, and this has prompted the development of alternative methods. For example, the inclusion of spin polarization via response functions based on a spin-restricted reference state (R-U approach) was developed by Fernandez et al.^33^ in 1992 and further extended by Rinkevicius et al.^34^ in 2004. The robustness of the R–U approach comes at the expense of losing a chemically intuitive and transparent connection to the MO theory and a straightforward visualization of the polarized spin density. Therefore, in our analysis, we stick to the unrestricted open-shell scalar-relativistic KS calculation, under the strict condition of a spin-contamination limit of Δ⟨*S*^2^⟩ < 0.01, which is valid for the electronic doublets studied in this work.

We have previously investigated a series of ruthenium(III) coordination compounds and found several ways in which the hyperfine interaction is dependent on the molecular arrangement^35^ and the substituent effects in the ligand fragments.^36,37^ Here, we investigate the FC mechanism of the hyperfine interaction between ^13^C and ^15^N atoms in an *N*-methylen-4-methylpyridinium (NMMP) radical model and extend our findings to a series of *trans*-[Ru^III^(NH_3_)_2_Cl_2_(4-methyl-pyridine)X] compounds. Initially, we examine the NMMP radical, where we alter the transmission mechanisms by rotating the methylene group. This manipulation allows for a clear distinction between the π-conjugation and σ-hyperconjugation delocalization pathways. Subsequently, we demonstrate how the *trans*-substituents in octahedral Ru(III) compounds influence the electron-spin distribution around the metal center as well as the nature of the metal–ligand bonds and consequent hyperfine interactions in the pyridine ligand. We delineate the delocalization and polarization mechanisms operating in both π and σ space, and elucidate how the π and σ coupling pathways synergistically enhance or attenuate the HFCCs for individual atoms.

## 2 Results and Discussion

Spin-transmission mechanisms are conventionally broken down into two components: spin delocalization and spin polarization. This framework is employed and further refined throughout the current study, as outlined below. By the *spin delocalization* contribution to the ligand HFC, we denote either the spin density at the ligand nucleus or the spin population of a particular ligand AO (depending on the context), obtained from a restricted open-shell calculation. In the existing literature, spin delocalization is commonly defined as the distribution of spin density resulting from the singly occupied molecular orbital (SOMO) at the unrestricted level of theory. This definition includes the response of the SOMO to the spin polarization of lower-lying MOs, whereas, in our approach, the SOMO response is included only within the valence-shell spin polarization. Regarding the aromatic ligand present in our systems, we distinguish between π-*conjugation* and σ-*hyperconjugation* delocalization mechanisms. A spin population of the ligand AO is assigned to a π-conjugation or a σ-hyperconjugation when the AO is antisymmetric or symmetric with respect to the ligand plane, respectively.

Lifting the restriction of identical spatial parts of α and β spin enables the *spin polarization* process to redistribute the electron density by allowing the class of α-spin orbitals to maximize exchange interactions with the SOMO. Classical Coulomb repulsions, electron–nucleus attractions, and kinetic energy terms must be optimized as well, which leads to a redistribution also within the class of β-spin orbitals. In TM complexes, spin polarization typically accumulates an additional α-spin population in the d (if symmetry-allowed, also s) orbitals of the metal. The driving force for this spin rearrangement is the maximization of the exchange interaction between the electrons of like spin, which is also the substance of Hund’s rule for maximizing total electron spin. The accumulation of like spin on the metal via spin polarization is thus assigned in schematic representations of spin-polarization pathways as a “Hund” or “exchange” component of the mechanism.

Spin polarization involves an additional accumulation of α-spin density at atoms contributing to the SOMO (in spin-restricted delocalization) thus resulting in an excess of β-spin density at their neighbors. This can be *neighbor-atom polarization* or *resonance polarization*, in both σ and π space, *vide infra*.

**Figure 1:**
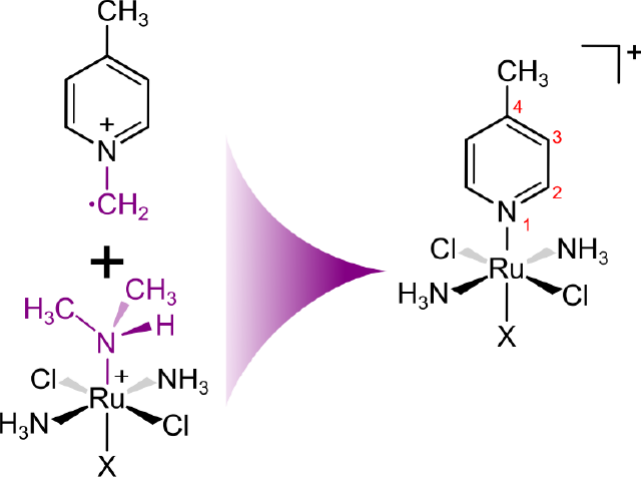
Structures of [Ru^III^(NH_3_)_2_Cl_2_(NHMe_2_)X]^+^ and *N*-methylen-4-methylpyridinium radical (NMMP) considered as building blocks of *trans*-[Ru^III^(NH_3_)_2_Cl_2_(4-Me-pyridine)X]^+^.

To illustrate the mechanisms and consequences of spin polarization and delocalization, we resort first to the systems [Ru^III^(NH_3_)_2_Cl_2_(DMSO)(NHR_2_)]^+^ (ruthenium coordination sphere) and the *N*-methylen-4-methylpyridinium radical (NMMP, pyridine ligand). These models represent two building blocks of [Ru^III^(NH_3_)_2_Cl_2_(DMSO)(4-Me-pyridine)]^+^ and analogous compounds, as depicted in Figure 1.

### 2.1 Mechanisms of Spin Transmission—Model Systems

#### 2.1.1 Neighbor-Atom σ-Polarization Mechanism

**Figure 2:**
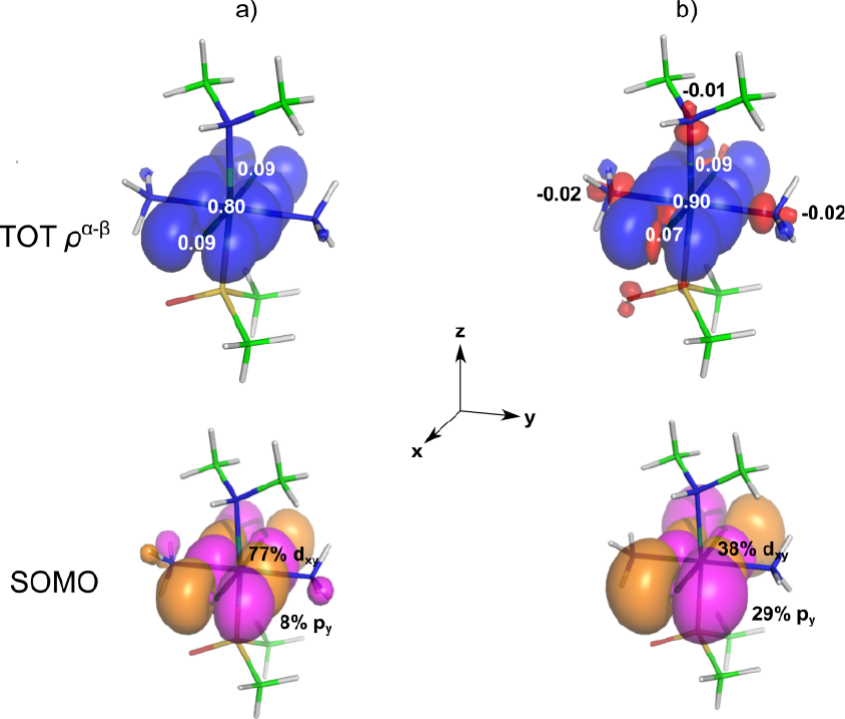
Visualization of the total spin density (top, α in blue, β in red, isovalue 0.0001 au) with gross Mulliken atomic spin populations^38^ and the SOMO with AOs contributions (bottom, isovalue 0.04 au, Löwdin analysis)^39^ for [Ru^III^(NH_3_)_2_Cl_2_(DMSO)(NHMe_2_)]^+^ calculated by using (a) spin-restricted and (b) spin-unrestricted formalism (PBE0/TZVPP).

To explore the role of the neighbor-atom σ-polarization mechanism independently (without involving the ground-state spin delocalization to the nitrogen ligand), we analyzed the model cation [Ru^III^(NH_3_)_2_Cl_2_(DMSO)(NHMe_2_)]^+^. This model demonstrates the transmission of spin density from the ruthenium to the nitrogen atom of the NH(CH_3_)_2_ group solely through a single-step spin polarization process as shown in Figure 2.

As a manifestation of Hund’s rule, the presence of α-spin density in the SOMO at the central metal atom (delocalized to Cl atoms, as shown in Figure 2a) induces α-polarization of the bonding orbitals at the ruthenium atom. This accumulation of α-spin density at the metal results in an excess of β-spin density at the neighboring bonded atoms (observed at all three nitrogens). In theory, this σ polarization propagates further to the next-neighbor atoms with the inverted sign because of Hund’s rule and the Pauli exclusion principle. However, because σ bonds have limited ability to spin-polarize their surrounding (due to their localized σ character), this mechanism is highly inefficient and barely noticeable at longer bond distances (such as the carbon atoms of the NH(CH_3_)_2_ group). Clearly, the long-range spin transmission observed in aromatic compounds originates from the different mechanisms discussed in the subsequent section.

#### 2.1.2 Delocalization Mechanisms

Spin delocalization can be linked to the electronic effects that underlie organic electronic structure and reactivity.^40^ In aromatic systems, π*-conjugation* is a familiar phenomenon, evident in the substituent mesomeric effects. However, σ*-hyperconjugation* also plays an important role,^41^ particularly in systems with nonplanar attachments to aromatic cores or in aliphatic systems. The σ-hyperconjugation mechanism has been used to interpret HFC pathways,^42^ spin-crossover,^43^ and exchange coupling^44^ in TM complexes. The mechanisms associated with π*-conjugation* and σ*-hyperconjugation* phenomena are examined and discussed in the subsequent subsections, focusing on two rotational states of the pyridinium radical model system.

##### 2.1.2.1 π-Conjugation Delocalization

**Figure 3:**
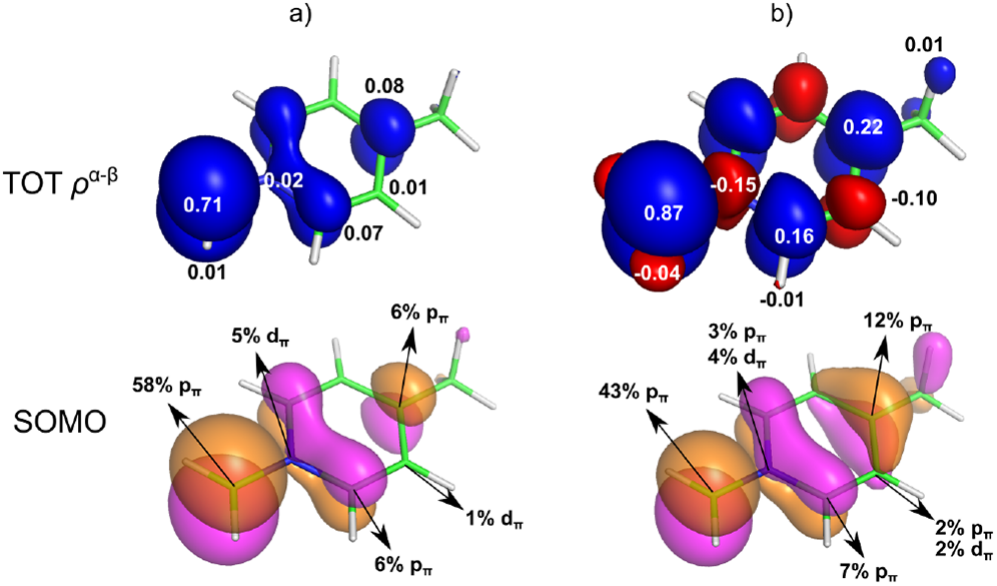
Visualization of the total spin density (top, α in blue, β in red, isovalue 0.0001 au) with gross Mulliken atomic spin populations and the SOMO with AOs contributions (bottom, isovalue, 0.04 au, Löwdin analysis) for a planar conformation (equilibrium, EQ) of *N*-methylen-4-methylpyridinium radical calculated by using the (a) spin-restricted and (b) spin-unrestricted formalism (PBE0/TZVPP). Note that the π space is perpendicular to the plane of the pyridine ring.

The distribution of the α-spin density in the equilibrium (EQ) conformation of the *N*-methylen-4-methylpyridinium radical (NMMP) is exemplified in Figure 3. Spin density is transmitted from the paramagnetic CH_2_ center onto the *ortho* and *para* carbon atoms in the SOMO, with the resulting spin populations for individual atoms calculated by using the spin-restricted formalism shown in Figure 3a. This delocalized α-spin density at the *ortho* (C2) and *para* (C4) positions intensifies in the spin-unrestricted calculation (Figure 3b), as it pulls the α-density from their surroundings via the spin-polarization (exchange) mechanism described above, thereby leaving the β-spin density at the neighboring atoms in the *ipso* (N1) and *meta* (C3) positions. In contrast to the inefficient consecutive neighbor-atom polarization discussed above, this *resonance polarization* (e.g., at C3) stems from the delocalized spin density (at C2 and C4). Note in passing a spin polarization of the hydrogen atoms of the CH_2_ group, although this will not be further elaborated, as it falls within the concept of neighbor-atom spin polarization discussed in the previous section.

##### 2.1.2.2 σ-Hyperconjugation Delocalization

**Figure 4:**
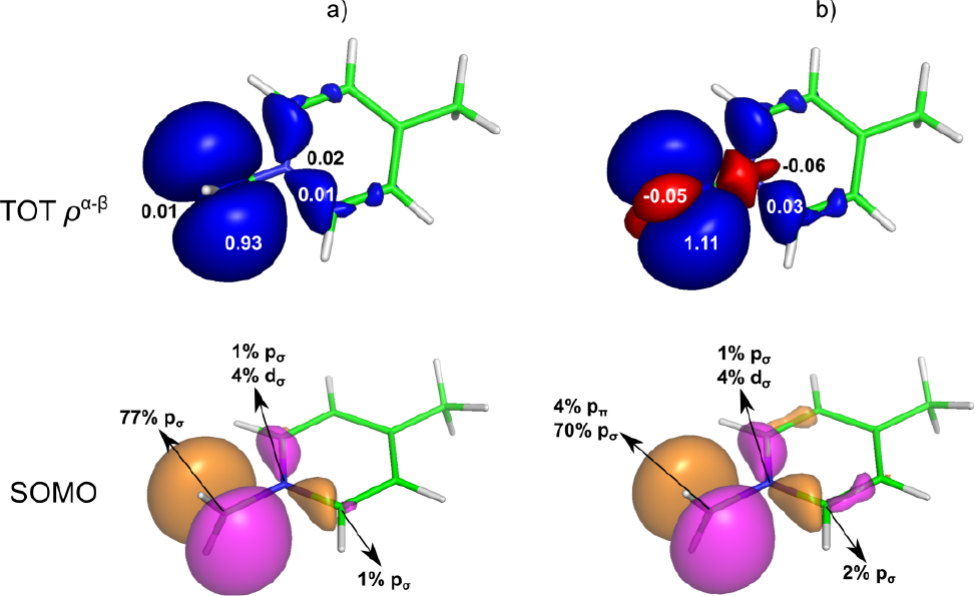
Visualization of the total spin density (top, α in blue, β in red, isovalue 0.0001 au) with gross Mulliken atomic spin populations and the SOMO with AO contributions (bottom, isovalue 0.04 au, Löwdin analysis) for a 90°-distorted conformation (transition state, TS) of the NMMP calculated by using the (a) spin-restricted and (b) spin-unrestricted formalism (PBE0/TZVPP). Note that the σ space is in the plane of the pyridine ring.

To quench the π-conjugation mechanism in the NMMP radical, we examined this system in its transition state (TS) obtained by rotating the CH_2_–N bond by 90°. In this π-quenched scenario, the concentrated α-spin density residing on the CH_2_ center is delocalized by the σ-hyperconjugation mechanism predominantly to the *ortho* positions C2 in σ-space, as illustrated by the spin-restricted calculation in Figure 4a. Additionally, to a lesser extent, the hyperconjugation mechanism also transmits the α-spin density to the C2–C3 bond and the *meta* carbon (C3). This distribution reflects the coefficients of the C2 and C3 atoms in SOMO, as shown in Figure 4a. In the spin-unrestricted approach, the σ-delocalized α-spin density at C2, along with that present at the CH_2_ group, induces the β-spin density in the σ-space of the *ipso* atom N1, Figure 4b.

#### 2.1.3 Altering π and σ Pathways in the NMMP Model

**Figure 5:**
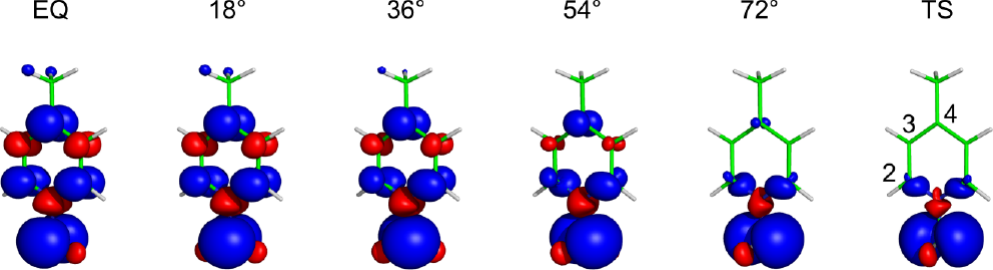
Visualization of the spin density (α in blue, β in red, isovalue 0.0001 au) in the NMMP radical for selected conformations (dihedral angle H–**C1–N1**–C2) obtained at the unrestricted DFT level by the rigid rotation of the H_2_C–N bond.

**Figure 6:**
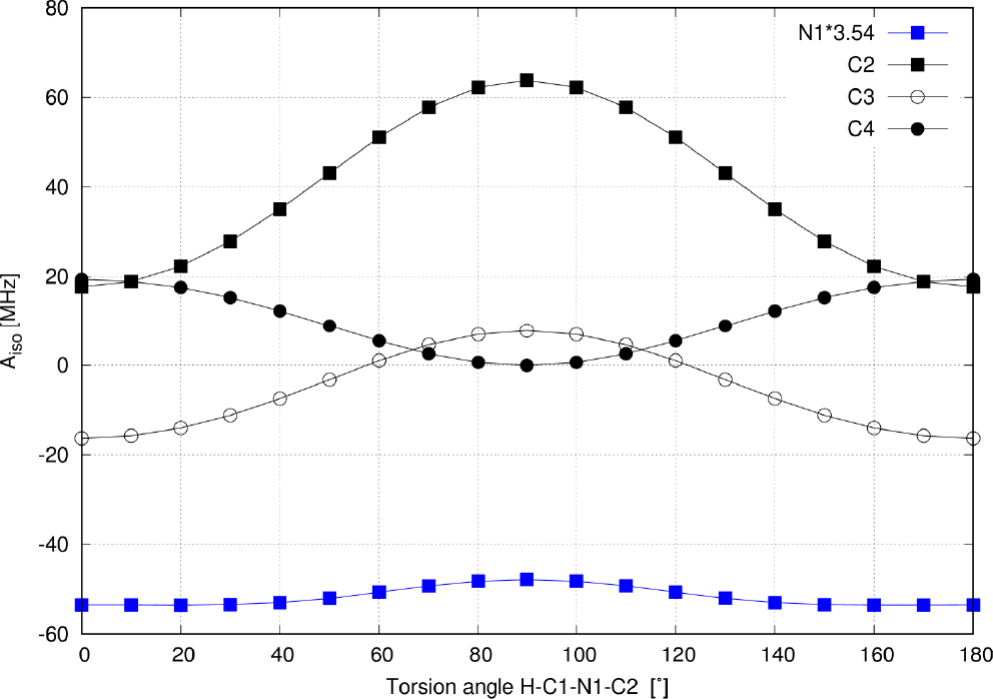
Dependence of the *A*_iso_ values (in MHz) for atoms N1, C2, C3, and C4 on the dihedral angle H–C1–N1–C2–rigid scan at a C1–N1 distance of 135 pm. Note the alternating π-spin density in the EQ (β at N1, C3 and α at C2, C4) and the induction of σ-spin density in the TS (β at N1, α at C2).

It should be noted that the bond distance CH_2_–N1 is different for the equilibrium geometry (EQ, 135 pm) and the geometry-optimized transition state (143 pm). Although this bond alteration does not change the transmission mechanisms described above, it can influence the bond covalency and the magnitude of the spin delocalization and polarization associated with it, as well as the resulting FC contribution to the HFC for the pyridine ligand atoms. Therefore, we performed a two-dimensional scan to systematically map the effects of the bond distance (covalency) and conformation (conjugation, hyperconjugation) on the HFCCs; see Figure S1.^45^ Indeed, the shorter interatomic distance with more efficient electron sharing is clearly reflected in the more effective spin delocalization and polarization of the aromatic ligand and the larger absolute values of the HFCCs. The distribution of spin density calculated for several torsion angles between the EQ and TS – all with the equilibrium CH_2_–N1 distance of 135 pm – is shown in Figure 5, and the corresponding isotropic HFCCs (*A*_iso_) are shown in Figure 6.

The most transparent behavior is observed for atom C4. The largest value of *A*_iso_(C4) is calculated for the EQ structure (+23 MHz) with efficient spin transmission via delocalization in the π-space from the CH_2_ group to C4, as shown in Figure 6 and Table S1. The conformational change (rotation) induces a significant drop in the *A*_iso_(C4) value because of quenching of the π-conjugation delocalization (vanishing in the TS).

If the *A*_iso_ for the C3 atom were driven by a mechanism involving exclusively delocalization in the π-space analogous to that discussed above for C4, its magnitude should drop during the rotation from the negative value of −18 MHz for the EQ structure and vanish in the TS (see red lobes at C3 in Figure 5). However, a vanishingly small *A*_iso_(C3) is observed already for the gauche conformation (close to 60 degrees) and a positive value of +6 MHz is calculated for the TS. This confirms the role played by the alternative σ-hyperconjugation transmission pathway, Figure 4b. In the planar EQ geometry, the SOMO is composed exclusively from the out-of-plane *p*_π_ of ligand atoms, including C3. In contrast, the in-plane SOMO in the distorted TS geometry consists partly of the in-plane *p*_σ_ AOs of C2 and C3. The two mechanisms give opposite signs of the HFCC, and thus their mutual compensation in the gauche conformation results in a vanishing *A*_iso_(C3).

The *A*_iso_(C2) = +21 MHz in the EQ state (0°) is mostly contributed by the π-conjugative delocalization pathway.^16^ Upon rotating the C–N bond, the π polarization starts to be less effective due to the unfavorable symmetry of the MOs. However, the *A*_iso_(C2) value rises to reach its maximum of +51 MHz in the TS! This is in clear contrast with the total spin population at the C2 atom, which drops significantly during this rotation (Figures S2 and S3). As expected, this is paralleled by a decrease in the ρ_π_ spin population at C2. Such behavior suggests that σ-hyperconjugative transmission governs the total *A*_iso_(C2) value in the TS. The hyperconjugation interaction analyzed by the NBO approach indicates two mechanisms. The α-spin density is propagated to the ligand by the most important and efficient hyperconjugation *n*(*p*)_α_ → N1=C2_α_* interaction of α-spin orbitals. In parallel, complementary back-hyperconjugation N1=C2_β_ → *n*(*p*)_β_* interaction between β-spin orbitals also leaves the overabundance of α-spin density at the N1=C2 bond. It should be highlighted that this σ-hyperconjugation transmission of spin density to the probed nucleus is significantly more efficient because of the direct involvement of the ligand *s* orbital in the SOMO.

In completing the picture for the pyridine ligand, N1 is less spin-polarized in the TS because π-space delocalization is missing from this conformation along with neighbor-atom polarization from the C2 atom linked to it.

To summarize our observations for the NMMP model, in EQ (0°), the α-spin is transmitted via the efficient π-delocalization channel complemented by the neighbor-atom and resonance spin polarizations (Hund’s rule), resulting in alternating signs of the ρ_π_ spin populations at the individual atoms of the pyridine ring, Figure 3b. In contrast, the π-transmission pathway is quenched in the TS (90°) and the α-spin density – now more concentrated and squeezed on the H_2_C fragment – starts to propagate via the σ channel, Figure 4b. The total pyridine spin population obtained at the restricted DFT level is 0.27 in the EQ and 0.05 in the TS. Despite approximately 5-fold more efficient delocalization via π in the EQ, the hyperfine coupling that originates in the π transmission can be smaller (compared to σ transmission) because the additional spin polarization of the ligand s-orbital is required to reach the atomic nucleus in the Fermi-contact mechanism (e.g., *A*_iso_(C2) is 21 MHz in EQ but 52 MHz in TS).

### 2.2 Metal–Ligand Bond and Trans Substituent in Ru(III) Compounds

**Figure 7:**
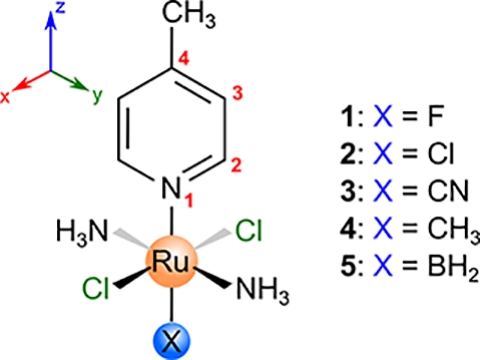
Structure and atom numbering scheme for compounds **1**–**5** highlighting the *trans*-ligand (X). Note the orientation of the Cartesian coordinate system with the Ru–N bond as the *z* axis.

After interpreting the spin-transmission mechanisms for the Ru(III) model and the NMMP system, we turned our attention to octahedral Ru(III) compounds with the general structure *trans*-[Ru^III^(NH_3_)_2_Cl_2_(4-Me-pyridine)X], Figure 7.

**Figure 8:**
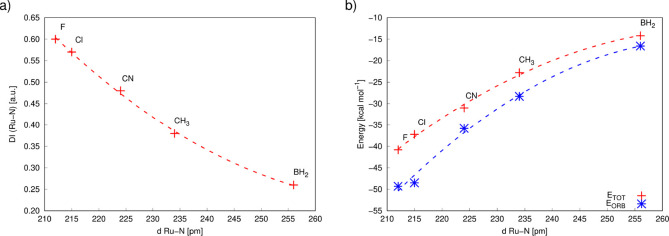
Correlation of the Ru–N1 distance (in pm) with (a) the QTAIM delocalization index (DI, Ru ↔ N in au) and (b) the interaction energy from the energy decomposition analysis (EDA, in kcal mol^–1^) for compounds **1**–**5**. For numerical data, see Table S2. Data points were fitted using quadratic interpolation to highlight the observed dependence.

The character of the Ru–N bond in compounds **1**–**5** is notably altered by the *trans*-ligand, with its effect (F < Cl < CN < Me < BH_2_) reflected in nonlinear inverse correlations between the Ru–N bond length and the Ru–N delocalization index^46,47^ (Figure 8a) or interaction energy^48^ (Figure 8b).

The weak Ru–F bond in compound **1** enables the formation of a strong covalent Ru–N1 bond in the *trans* position, characterized by a large DI of 0.6 au and an interaction energy of −40 kcal/mol. This phenomenon is associated with the structural *trans*-effect for the F–Ru–N1 fragment, where the two ligand atoms compete in binding to the central metal atom through a single metal d-orbital.^49,50^ In contrast, the formation of a strong covalent Ru–B bond in compound **5** significantly reduces the efficiency of electron sharing (covalency) between the ruthenium and the nitrogen atom in the *trans* position, evidenced by a small DI of 0.26 au and an interaction energy of −15 kcal/mol. This difference has notable implications for the magnitude of the spin-delocalization and resonance spin-polarization processes via the Ru–N1 bond to the pyridine ligand, as discussed further below.

#### 2.2.1 Modulation of Ligand HFCC by the Trans Ligand

The nature of *trans*-ligand X strongly influences the covalency of the Ru–N bond and the relative orientation (dihedral angle) between the Ru–Cl bonds and the plane of the pyridine ligand, which in turn affects the spread of spin density to the individual probed nuclei of pyridine. This spread is directly reflected in ligand HFCC, *A*_iso_.

**Figure 9:**
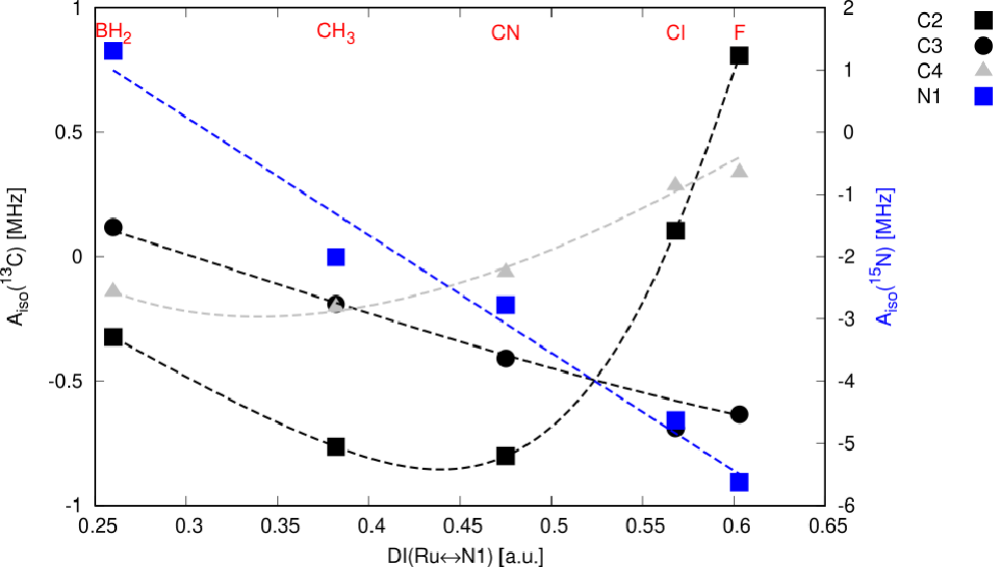
Dependence of *A*_iso_ (N1, C2, C3, and C4) on DI (Ru ↔ N) in compounds **1**–**5**. Note the approximately linear dependence for N1 and the highly nonlinear behavior for C2 and C4 (trend lines were obtained as cspline curves).

Generally, HFCCs for ligand atoms are expected to be relatively small in systems with an ionic Ru–N bond (larger interatomic distance and limited electron sharing) and larger in systems with a more covalent Ru–N bond (more efficient electron sharing). The dependencies of the HFCCs on the DI(Ru ↔ N) for selected ligand atoms N1, C2, C3, and C4 in compounds **1**–**5** are shown in Figure 9 (for hydrogen atoms H2 and H3, refer to Table S3).

Therefore, one would anticipate a small HFCC for atom N1 in compound **5** (X = BH_2_) with the longest Ru–N bond. Conversely, the magnitude of the HFCC for N1 should increase in compound **1** (X = F) with the shortest and most covalent Ru–N bond. While this general trend is approximately observed, a change in the sign of *A*_iso_(N1) indicates a shift in the spin-transmission mechanism. This is evident from the correlation between *A*_iso_ and DI, particularly for atoms C2 and C4, as shown in Figure 9.

Calculation of the HFCCs at the four-component relativistic DFT level has revealed that the Fermi-contact mechanism governs hyperfine coupling for nitrogen and carbon atoms (Figure S4). This observation justifies our focus on the analysis and interpretation of the distribution of spin density and atomic spin populations to understand the revealed trends by analogy to our NMMP model (Section 2.1). Because the HFCC of atom N1, situated on the transmission path between the paramagnetic metal center and atoms C2 and C4, displays a relatively linear correlation with the distribution of spin density, explaining the nonlinearity observed for the C2 and C4 (Figure 9) necessitates consideration of alternative pathways of spin propagation independent of neighbor-atom polarization (according to Dirac’s model)^51^ and π-space delocalization. When clarifying this reliance in accordance with our NMMP model, one may consider that in the favorable conformation the N1–C2 bond directly contributes to SOMO via σ-hyperconjugation. In the subsequent section, we delve into the analysis of spin-transmission mechanisms in compounds **1** and **5**, which exhibit different behaviors as identified above (Figure 9).

### 2.3 Spin-Transmission Mechanisms in Compounds 1 and 5

#### 2.3.1 Spin Densities and Spin Populations

**Figure 10:**
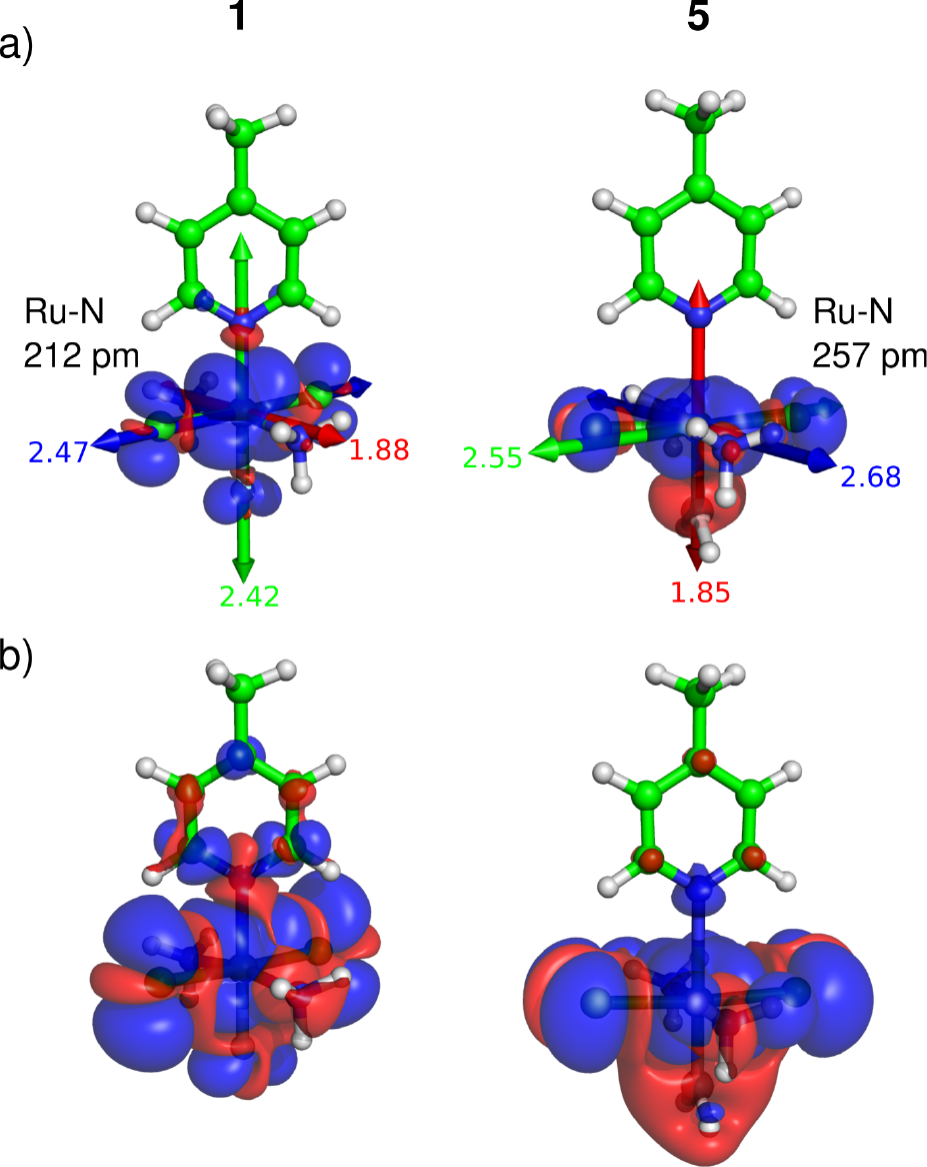
Total spin density (α in blue, β in red) in compounds **1** (X = F) and **5** (X = BH_2_) calculated for the relaxed structures at the 1c ZORA/PBE0/TZ2P level of theory. (a) Isosurface at 0.001 au highlighting the orientation of the SOMO and (b) isosurface at 0.0001 au showing the spin distribution in the pyridine moiety. The components of the g-tensor (calculated at the 2c SO-ZORA/PBE0/TZ2P level) are depicted by arrows (for the **g** tensors of compounds **1**–**5**, see Figure S6). Color labeling reflects the magnitude (red < green < blue); the smallest component shown in red is always perpendicular to the plane of the metal *d*-based SOMO.

In general, the propagation of spin density from the ruthenium center to the pyridine ligand is notably more efficient in compound **1** (X = F) compared to that in compound **5** (X = BH_2_), as shown in Figure 10. This behavior stems from the different spin-transmission mechanisms, as the change in the ligand not only alters the Ru–N distance and covalency but also triggers rearrangement of the electron configuration (refer to Figure S5). Consequently, the unpaired electron resides in a SOMO of different spatial symmetry relative to the Ru–N bond, Figure 10. Specifically, the unpaired electron is situated in a d_*xz*_-type molecular spin–orbital (MSO) in compound **1** (X = F), whereas in compound **5** (X = BH_2_), it occupies an equatorial d_*xy*_-based MSO, akin to NAMI analogs reported previously.^25,35,37,52^

The difference in the orientation of the SOMO between compounds **5** and **1** reflects distinct Ru–N bonding characteristics in these two compounds,^53−55^ and results in different transmission mechanisms and a reversed π-polarization of pyridine in these compounds (Figure 10). Note that the change in the Ru–N bond distance also leads to a variation in the orientation of the pyridine plane relative to that of the equatorial ligands (Cl and NH_3_). In compound **5** (X = BH_2_), the plane of the aromatic ring is almost exactly in an eclipsed conformation with respect to the equatorial Ru–Cl bonds, facilitated by a slightly longer Ru–N bond of approximately 257 pm. In contrast, the dihedral angle of H_3_N–Ru–N1–C2 in complex **1** (X = F) measures 58°, attributed to the interatomic repulsions arising from a shorter Ru–N1 bond of around 212 pm.

#### 2.3.2 Mechanisms in Compound 1

**Figure 11:**
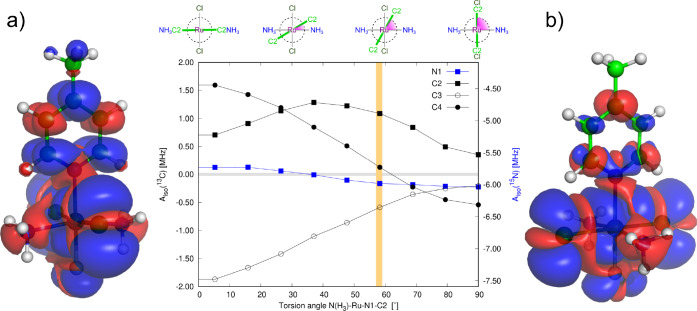
Dependence of the *A*_iso_ values of N1, C2, C3, and C4 (in MHz) on dihedral angle N–Ru–N1–C2 for compound **1**. The orange band depicts the equilibrium conformation. The distribution of spin density (α in blue, β in red, isovalue 0.0001 au) for the conformations of (a) 0° and (b) 90° is also shown. For an animated file of the conformation-dependent spin density, see the Supporting Information.

To systematically analyze delocalization and polarization mechanisms and transmission pathways in compound **1**, and to draw connections with observations made for the NMMP model, we first performed a relaxed conformation scan of the H_3_N–Ru–N1–C2 torsion, Figure 11. Subsequently, we calculated the electronic structure of compound **1** in its two extreme states (with a relaxed Ru–N bond), differing in the constrained orientation of the pyridine ring relative to the SOMO (at 90° and 0°, Figure 11). These two orientations resemble the situations for the two stationary states discussed in Section 2.1 (EQ and TS for the NMMP radical, respectively). However, unlike NMMP, the π-delocalization is not completely quenched in the eclipsed conformation of compound **1**; instead, it is redirected from the pyridine to the chlorine atoms, *vide infra*.

##### 2.3.2.1 Perpendicular (Out-of-Plane) Conformation (0°)

**Figure 12:**
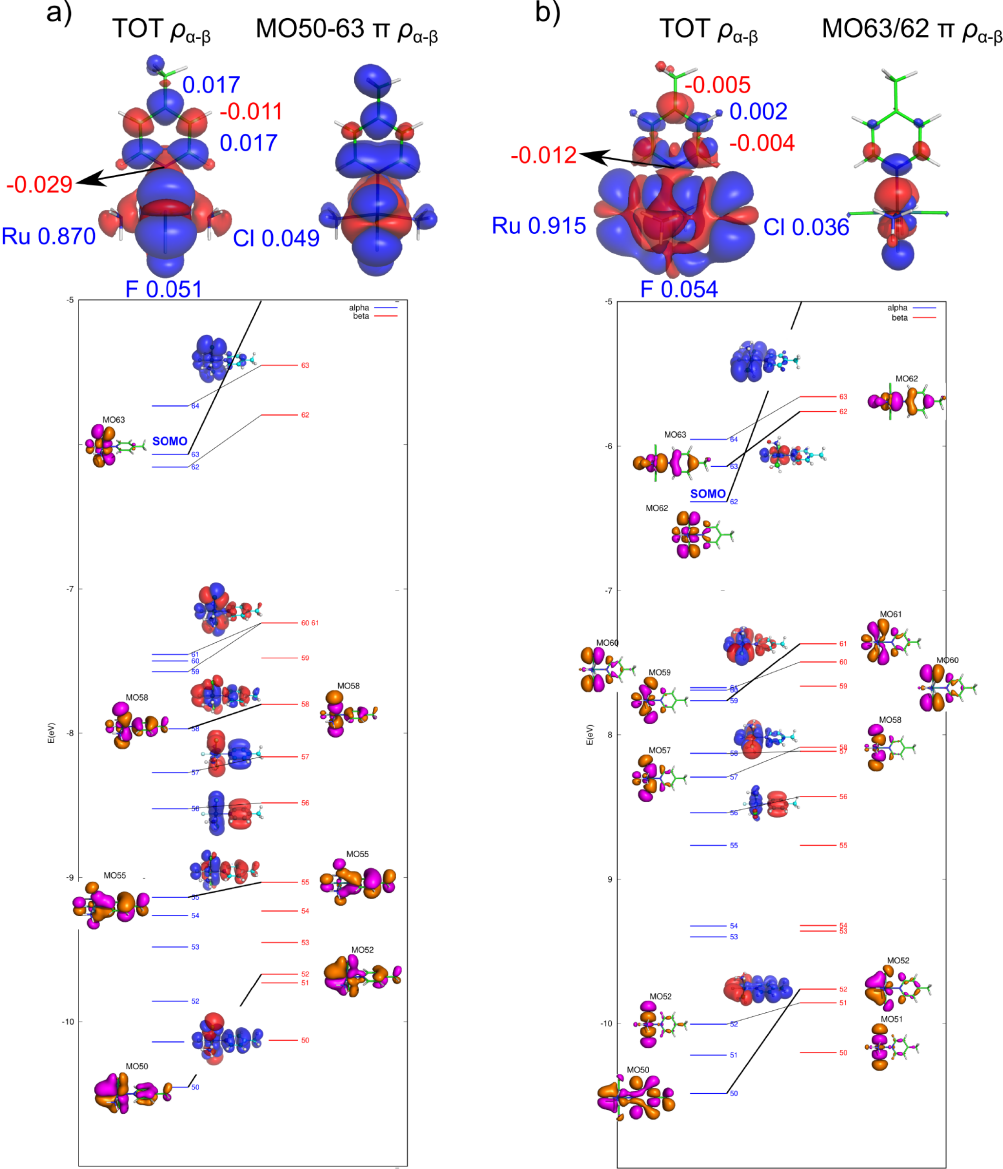
Visualization of the spin density (α in blue, β in red, isovalue 0.0001 au), gross Mulliken atomic spin populations, and diagrams of α and β MSOs for (a) the perpendicular and (b) the eclipsed conformations of the pyridine ring relative to the Cl–Ru–Cl bond (SOMO). The calculation was performed at the PBE0/def2TZVPP level using an unrestricted approach.

The metal d_*xz*_ AO interacts with *p*_*z*_ AOs at both chlorides to form  in the Cl–Ru–Cl fragment (MSO α-63 in Figure 12a). This MO is singly occupied (SOMO), and its symmetry (Figure 11) enables contributions from the out-of-plane AOs (π) of the pyridine atoms, as rationalized for the EQ state of the NMMP in Figure 3. Because of the π-delocalization of the SOMO to atoms C2 and C4, the restricted spin pattern at the pyridine ring resembles that obtained for the EQ of the NMMP. Analogous to the NMMP radical, resonance spin polarization on the spin-delocalized system results in the additional concentration of α-density at C2 and C4, leading to an overabundance of β-density at N1 and C3 (see MOs 55 and 58 and spin density in Figure 12a). Therefore, the total spin pattern at the pyridine ring is similar to that identified and discussed for the EQ state of NMMP, Figure 3b.

Similar to the NMMP radical, HFC is most transparent for the atom C4 in the *para* position, influenced exclusively by the π-transmission (spin population +0.017, *A*_iso_ +1.6 MHz). In contrast, C2 in the *ortho* position is influenced by the π-delocalization (α), which is however somewhat balanced by the neighbor-atom σ-polarization (β), resulting in the atomic spin population +0.017 and *A*_iso_ +0.7 MHz. This behavior is greatly affected by the rotation around the Ru–N bond, as shown in Figure 11.

##### 2.3.2.2 Eclipsed (In-Plane) Conformation (90°)

In the eclipsed conformation, the SOMO is contributed from the in-plane AOs of the pyridine atoms. This is reflected in the significant spin delocalization via the σ-hyperconjugation pathway; however, it is accompanied by secondary resonance polarization of the pyridine ligand in the π-space (Figure 11). It is worth noting that this secondary resonance polarization does not operate in the TS conformation of NMMP, where the *trans* ligand is absent at the paramagnetic center. The mechanism of this secondary resonance polarization via near-energy canonical MOs is elucidated in the following paragraph.

As demonstrated for the TS conformation of NMMP in Figure 4b, α-spin density is transmitted in the eclipsed conformation via the in-plane σ-hyperconjugation mechanism (MSO α-62 in Figure 12b). However, the presence of the F ligand in the position *trans* to the Ru–N bond enables an alternative transmission pathway in compound **1**. In addition to the dominant Cl–Ru–Cl fragment (Ru d_*xz*_ 45%, Cl p_*z*_ 44%), the α-spin density is delocalized via the Ru–F bond (F p_*x*_ 5%) in the SOMO of in-plane symmetry (MO α-62). Initially, this spin polarization affects MOs of the same symmetry because of the efficient electron-exchange interaction, as exemplified by MSOs α-50 and β-52. As a manifestation of Hund’s rule, the metal d_*xz*_ contributes 1% more to MSO α-50, resulting in polarization of the axial F and pyridine (note the significant contributions to C2, C3, and C4) ligands. A considerable β-spin polarization of the F atom in this MSO pair (with a 41% difference for F p_*x*_ between α-50 and β-52) induces the opposite (α-spin) polarization of F in the other two important MSO pairs, α-59−β-61 and α-63−β-62. Although the first MSO pair is responsible for the hyperconjugative polarization in the σ-space, enhancing the effect of the SOMO and α-50−β-52, the roles of the second pair is different. This MO (α-63−β-62) of π-symmetry has an SOMO neighbor and is polarized via electron exchange around the *trans* F atom (1.6% p_*y*_ predominance of α), resulting in an overabundance of β-density in the out-of-plane p_*y*_ of the appropriate symmetry at C2 (see the spin-density pattern in Figure 12b). Note that this resonance spin-transmission pathway, absent from the NMMP model, results in a spin pattern on the pyridine ring in the eclipsed conformation opposite those for the out-of-plane conformation of **1** and the EQ state of NMMP.

In contrast to the TS conformation of the NMMP radical, where the HFC for atom C4 in the *para* position is vanishingly small, in the eclipsed conformation of **1**, this atom is influenced by resonance π-polarization, resulting in an atomic spin population of −0.005 and *A*_iso_ −0.5 MHz. For atom C2, the counter effects of positive σ-hyperconjugative delocalization and negative resonance π-polarization yield an atomic spin population of −0.04 but *A*_iso_ = +0.4 MHz.

In the equilibrium (fully optimized) geometry of compound **1**, with the torsion angle close to gauche (58°, indicated by the orange line in Figure 11), both mechanisms analyzed above for the perpendicular and eclipsed conformations operate simultaneously, leading to the pattern shown in Figure 10. However, due to the larger efficiency of π-delocalization, the total spin-density pattern is evidently more similar to that of the perpendicular conformation.

#### 2.3.3 Mechanism in Compound 5

**Figure 13:**
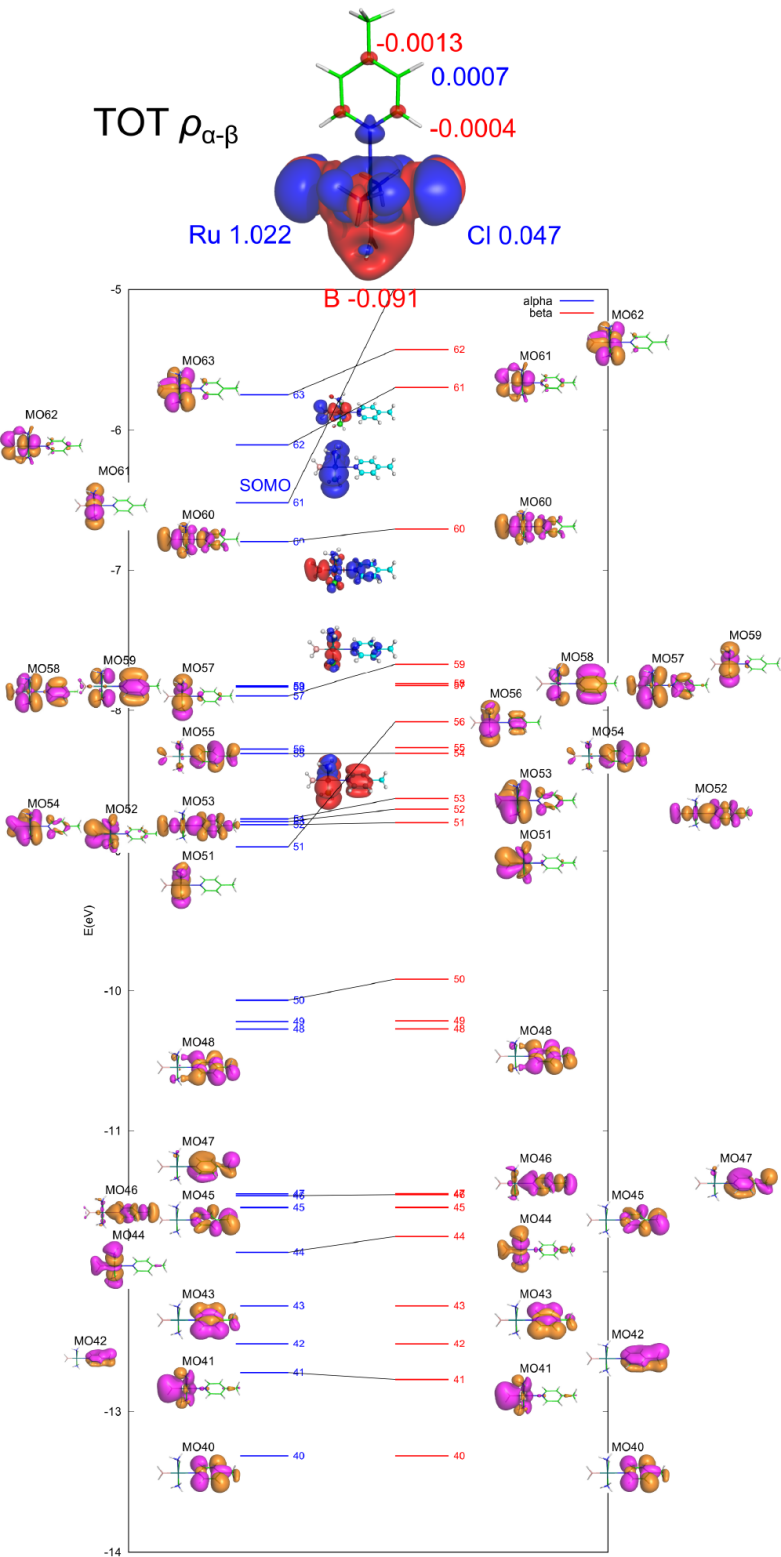
Visualization of the spin density (α in blue, β in red, isovalue 0.0001 au) and MSO diagram for compound 5. The calculation was performed at the PBE0/def2TZVPP level using an unrestricted approach.

In the molecular frame of compound **5** (Figure 10), the orientation of the SOMO (d_*xy*_-based) prevents efficient admixture of pyridine atoms (both in-plane and out-of-plane) in this MSO. Consequently, the SOMO is dominated by the Ru (d_*xy*_ 48%) and Cl (p_*y*_ 49%) atoms. Because of the symmetry-blocked delocalization of the α-spin density to the pyridine ligand, the concentration of α-spin at the ruthenium atom results in the polarization of the MOs with significant contributions from the metal AOs. Thus, the electron-exchange interaction polarizes MOs with the metal  and  character, adhering to Hund’s rule, thereby concentrating the α-density around the metal and β-density in the σ-space of the axial BH_2_ and equatorial chloride ligands. However, this neighbor-atom polarization is notably inefficient for the pyridine nitrogen atom because of the weak and long Ru–N bond (257 pm, see Figure 8), losing the competition with the strong axial (*trans*) BH_2_ ligand.^49,56,57^ This inherently limits the efficiency of polarization of the pyridine ligand. Additionally, the strong and covalent Ru–B bond (α Ru 69%, B 31%; β Ru 64%, B 36%) is formed predominantly by  on the side of ruthenium (s 14%, p 4%, d 82%). Therefore, the weaker Ru–N bond (Ru s 2%, p 95%, d 3%) is driven by p_*z*_–p_*z*_, which is β-polarized on the side of the Ru and α-polarized in the σ-space of the N atom (Figure 13). The secondary resonance α-polarization of the pyridine nitrogen and β-polarization of the *ortho* and *para* carbons via the π-type MSOs (α-62−β-61) is similar to that identified and discussed for the eclipsed conformation of compound **1**.

In summary, the distinct orientations and compositions of the SOMO in compounds **1** and **5** dictate varied mechanisms of spin-density transmission from the metal toward the pyridine ligand.

### 2.4 Resonance Polarization in Compound 6

**Figure 14:**
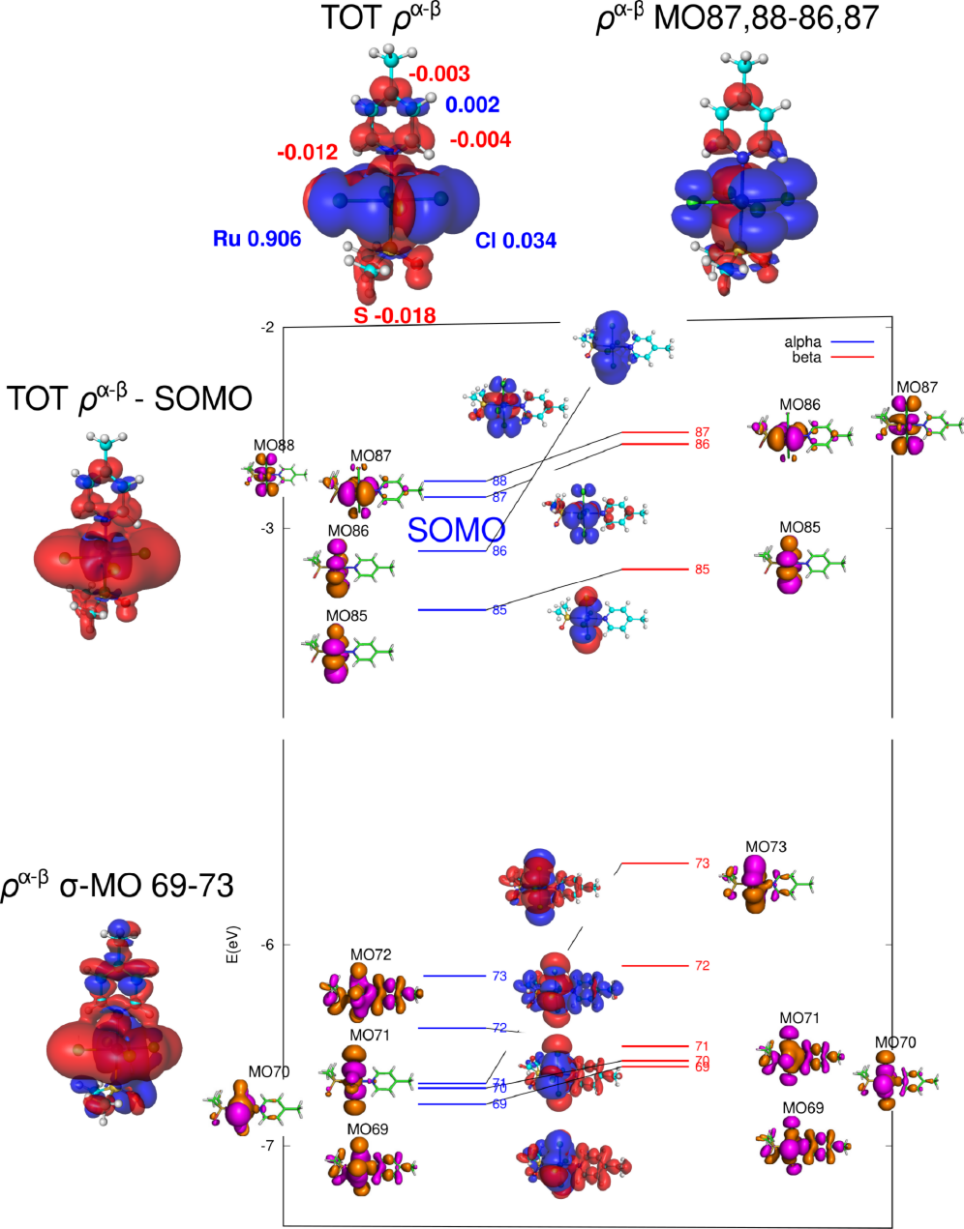
Visualization of the spin density (α in blue, β in red, isovalue 0.0001 au) and partial MSO diagram for compound **6**, *trans*-[Ru^III^Cl_4_(DMSO)(4-Me-pyridine)]^−^. The calculation was performed at the PBE0/def2TZVPP level using an unrestricted approach.

As the final step, we turn to negatively charged compound **6** with four chlorides at equatorial positions and a DMSO ligand in the axial *trans* position, as shown in Figure 14. This system serves as an analog of biologically significant compounds such as NAMI,^58,59^ [RuCl_4_(DMSO)(imid)]^−^, and KP1019,^60,61^ [RuCl_4_(indaz)_2_]^−^. Note that the Ru–N distance in this compound is short (211 pm) and comparable to that in compound **1**.

In compound **6**, the SOMO possesses d_*xy*_ symmetry and receives significant contributions from the lone pairs of electrons of the four Cl atoms. However, similar to the configuration of compound **5** discussed above, the pyridine atoms do not contribute to the SOMO at the spin-restricted level. Consequently, spin transmission can reach the atoms of the pyridine ligand only via a spin-polarization mechanism. Thus, neighbor-atom spin polarization leads to an overabundance of β-spin density in the σ-space of the axial DMSO and pyridine ligands (Ru–N and Ru–S bonds, Figure 14). The hyperconjugation interactions originating from the polarized Ru–N bond further propagate the β-density in the σ-space (lower-lying MOs of 69–73). Analogous to the eclipsed conformation of compound **1** and compound **5**, π-type MOs (α-87,88 and β-86,87) serve as the source of resonance β-polarization of C2 and C4. Therefore, the total spin-density pattern across the pyridine ligand in the equilibrium conformation of compound **6**, as shown in Figure 14, is governed by *resonance polarization* in both the σ and π spaces.

To demonstrate the relevance of our computational methodology, we present in Table 1 the calculated hyperfine ^13^C NMR shifts for compound **6**, which closely align with the experimental data. These values also reveal minor spin–orbit effects on the investigated ^13^C NMR shifts, while highlighting significant and disparate solvent effects on the shielding of individual carbon atoms of pyridine (cf. NMR shifts of C3 and C4 in vacuo and implicit solvent). These changes in hyperfine shielding are attributed to the efficacy of the individual σ- and π-resonance polarization pathways.^35^

**Table 1:** Calculated (ZORA/PBE0/TZ2P)^a^ and Experimental Hyperfine ^13^C NMR Shifts (in ppm)^35^ for Compound **6**

	calculated ZORA (vacuo)	calculated SO-ZORA (vacuo)^b^	calculated SO-ZORA (COSMO)^b^	experimental^b^
C2	–72	–84	**–75**	**–72**
C3	–5	–7	**–28**	**–25**
C4	–48	–52	**–28**	**–27**

a ZORA refers to the 1c calculation of the HFC tensor and the 2c calculation of the electronic **g** tensor; SO-ZORA refers to the 2c calculation of both **A** and **g** tensors.

b Ref.^35^

To summarize our observations for compounds **1**–**6**, we showed that in symmetry-allowed situations, the primary spin-transmission mechanism throughout the aromatic system is either π-conjugation or σ-hyperconjugation delocalization. These mechanisms are accompanied by neighbor-atom and resonance polarizations, which generally result in further accumulation of α-spin density at the atoms contributing to the SOMO, while retaining β-spin density at the neighboring atoms. However, in cases of symmetry-quenched or symmetry-redirected delocalization of the SOMO, the pyridine ligand can be weakly spin-polarized in the σ- and/or π-space through resonance polarization. This leads to an inverted spin pattern across the aromatic ring.

## 3 Conclusions

We conducted a comprehensive DFT investigation of isotropic hyperfine couplings (HFC) for aromatic pyridine ligands in prototypical octahedral TM complexes, with the aim to decipher spin-transmission mechanisms. Our approach integrates qualitative model-free analysis based on the total distribution of spin density with quantitative population analysis of canonical MOs. The pathways we described are connected to fundamental chemical concepts, such as delocalization and hyperconjugation. We show that π*-conjugation* delocalization from the metal d-orbital is the most effective mechanism, although σ*-hyperconjugation* delocalization proves to be surprisingly efficient as well and can even dominate the ligand HFC. This efficiency is attributed to the direct involvement of the ligand atomic s-orbital in the SOMO, which is crucial for the Fermi-contact mechanism of the HFCC. The well-known alternating spin-density pattern on the aromatic ligand is achieved through neighbor-atom and resonance polarizations.

Generally, delocalization from the metal to the aromatic ligand and the related ligand polarization are noticeably weaker for the σ as compared to the π arrangement of the pyridine, in both the organic radial and the TM complex. The polarization of the aromatic ligand in the σ arrangement is approximately five times smaller. We highlight that although π delocalization is completely quenched by rotation from the π to the σ conformation of the organic radical, in octahedral TM complexes, it is merely redirected from pyridine to the equatorial ligands. We separated the σ and π transmissions using rotation models but also demonstrated that both conformation-dependent pathways can contribute to the HFC in the relaxed (geometry-optimized) systems.

Finally, we establish a relationship between the HFC and the metal–ligand bond properties described by bonding parameters and showcase the effect of a *trans* ligand on the HFC to formulate the concept of *hyperfine trans effect*. Our findings enrich the current understanding of spin-transmission mechanisms and offer valuable insights for practitioners in the fields of EPR and NMR to interpret their experimental observations in paramagnetic molecular systems and materials of varying complexities.

## 4 Methods

### 4.1 Molecular Geometry

All starting structures were optimized in the Turbomole 7.00 program^62^ using a previously calibrated DFT approach,^35,63,64^ utilizing the PBE0 functional^65^ and the def2-TZVPP basis set^66^ with the corresponding def2-ECP^67^ relativistic effective core potential for the ruthenium atom. All calculations were performed in a vacuum. Constrained optimizations and the relaxed scan of compound **1** were carried out using Orca 5.0 software^68^ (PBE0/def2-TZVPP/ECP).

### 4.2 EPR Parameters

EPR parameters were calculated with the program Orca 5.0 program (PBE0/def2-TZVPP/ECP). For the fully optimized models, hyperfine coupling constants were also calculated using ZORA (scalar-relativistic and spin–orbit, collinear approximation) as implemented in the ADF package.^69^ The PBE0 functional was used together with a TZ2P basis set. Collinear two-component ZORA calculation also provided the values of the g-tensors summarized for compounds **1**–**6** in Figure S6.

### 4.3 Spin Density and Population Analysis

Atomic gross spin populations were determined at both the restricted and unrestricted level using Mulliken population analysis.^38^ Canonical KS MSOs were analyzed using Löwdin population analysis.^39^ Corresponding spin polarizations of pairs of MSOs were calculated by using the cubman tool of the Gaussian package. The spin contamination in our calculations of electronic doublet systems with ⟨*S*^2^⟩ = 0.75 amounts to maximum 0.009 for compound **5**, which is considered marginal. The corresponding numbers are summarized in Figure S6.

### 4.4 Bonding Analysis

Energy decomposition analysis (EDA)^48^ with natural orbitals for chemical valence (NOCV)^70,71^ was performed at the ZORA/PBE0/TZ2P level by using the ADF package. Delocalization indices (DIs)^46,47^ were calculated in AIMAll^72^ using PBE0/def2-TZVPP/def2-ECP wave functions and the Gaussian 09 program.^73^

## Supporting Information Available

Additional calculated data; 2D plots of the dependences of *A*_iso_ values on the H_2_C–N1 distance (Figure S1); values of *A*_iso_ and spin populations in p_π_ AOs (Table S1); total spin population for atoms N1, C2, C3, and C4 (Figure S2); p_*z*_ and total-p_*z*_ spin populations for atoms N1, C2, C3, and C4 (Figure S3); interatomic distance, delocalization index, and bond energy (Table S2); isotropic *g*-value and HFC (Table S3); correlation of the Fermi-contact contribution and total HFCC (Figure S4); energy diagram of the highest occupied Kohn–Sham MSOs (Figure S5); and visualization of spin density (Figure S6) (PDF)
Visualization of rotation-dependent distribution of spin density for compound **1** (MP4)
Cube files of spin densities for compounds **1**, **5**, and **6** (ZIP)
