# Supplementary material for: Mechanisms of Ligand Hyperfine Coupling in Transition-Metal Complexes: σ and π Transmission Pathways
Source: Inorg Chem. 2024 May 1;63(19):8580–92. doi: 10.1021/acs.inorgchem.3c04425 (PMC11094796; doi:10.1021/acs.inorgchem.3c04425)
Supplement: Supplementary file 1 — ic3c04425_si_001.pdf [file ic3c04425_si_001.pdf]

Supporting Information for

Mechanisms of Ligand Hyperfine Coupling in Transition-Metal Complexes: σ and π Transmission Pathways

Jan Novotný,*[†,‡] Markéta Munzarová,[‡] and Radek Marek*[,†,‡]

[†] *CEITEC – Central European Institute of Technology, Masaryk University, Kamenice 5, CZ – 62500 Brno, Czechia*

[‡] *Department of Chemistry, Faculty of Science, Masaryk University, Kamenice 5, CZ – 62500 Brno, Czechia*

Email: jan.novotny@ceitec.muni.cz (J.N.), radek.marek@ceitec.muni.cz (R.M.)

SUPPLEMENTARY DATA AND FIGURES

N-methylen-4-methylpyridinium radical

First, the role of covalency was roughly estimated by varying the interatomic distance between the nitrogen atom of the pyridine skeleton and the carbon atom of the $H_2C\cdot$ group. Second, rotating the *p*-type SOMO located at the methylene group was intended to imitate approximately the situation of altering the symmetry of a *d*-based SOMO in transition-metal complexes, *vide infra*. The resulting 2D scan performed for the $H_2C\cdot$–N1 bond shows a significant dependence of the A_{iso} value on both the interatomic distance and the torsion angle, **Figure S1**.

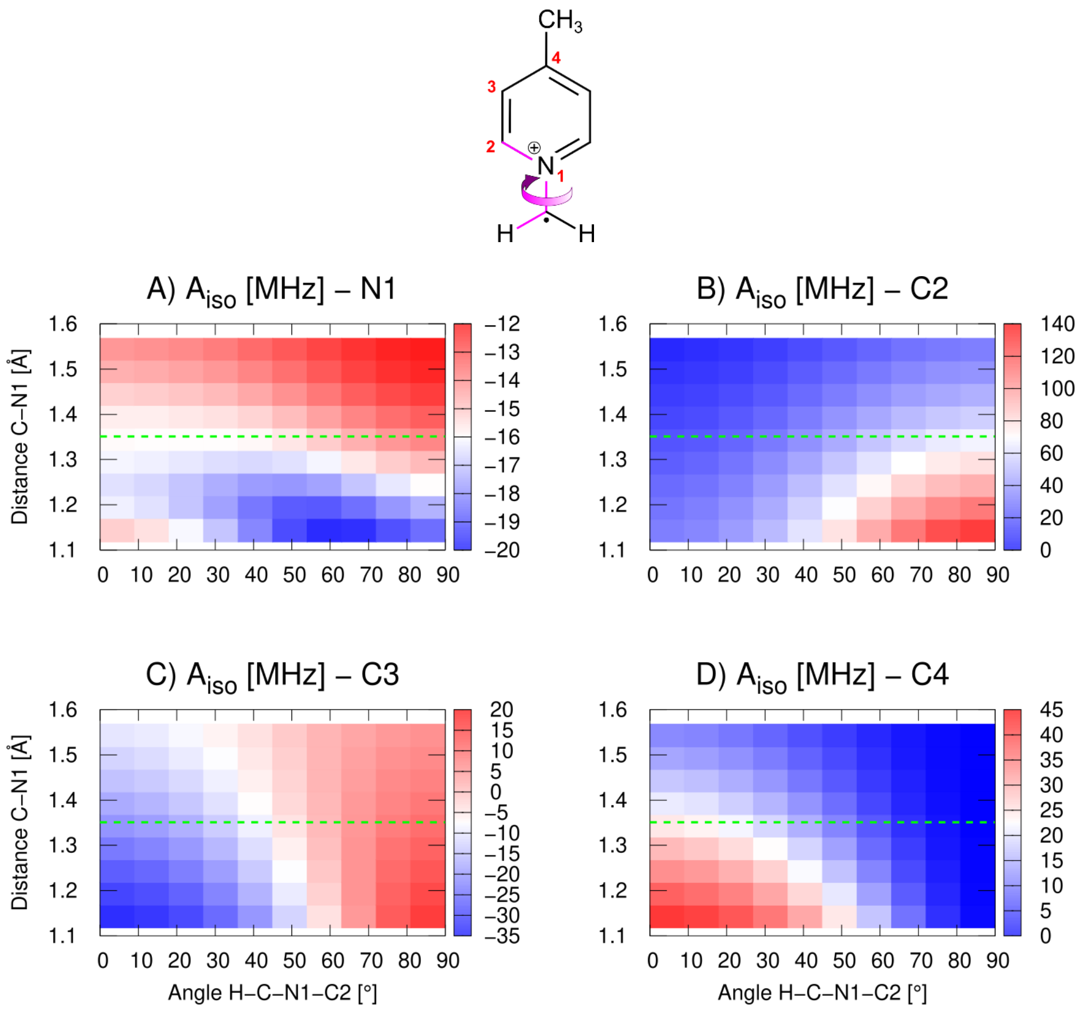

Figure S1. 2D plots of the dependences of A_{iso} values on the H_2C–N1 distance (in Å) and the conformation (H-C-N1-C2 angle in degrees) for atoms A) N1, B) C2, C) C3, and D) C4 in *N*-methylen-4-methylpyridinium radical. Calculations were performed at the ZORA/PBE0/TZ2P level of theory. The equilibrium $H_2C\cdot$-N1 distance (1.35 Å) is shown with a green line.

Table S1. Values of A_{iso} and spin populations in p_π AOs for selected atoms of pyridine in *N*-methylen-4-methylpyridinium radical (NMMP) as a function of the dihedral angle H-C-N1-C2.

	A_{iso} [MHz]						p_π spin population [a.u.]					
atom\dih.	0	45	60	70	80	90	0	45	60	70	80	90
**N1**	-15.95	-14.06	-12.82	-12.08	-11.77	-12.63	-0.0949	-0.0748	-0.0561	-0.0402	-0.0233	-0.0094
**C2**	+21.03	+26.17	+31.87	+37.28	+43.78	+50.81	+0.1404	+0.0968	+0.0666	+0.0443	+0.0226	+0.0053
**C3**	-17.78	-10.82	-5.47	-1.26	+2.99	+6.62	-0.0810	-0.0577	-0.0403	-0.0270	-0.0140	-0.0036
**H2**	-14.09	-9.84	-6.60	-4.14	-1.73	+0.20	*+0.1404*	*+0.0968*	*+0.0666*	*+0.0443*	*+0.0226*	*+0.0053*
**H3**	+6.29	+4.79	+3.78	+2.99	+2.26	+1.70	*-0.0810*	*-0.0577*	*-0.0403*	*-0.0270*	*-0.0140*	*-0.0036*
**C4**	+22.75	+15.58	+10.46	+6.68	+3.01	+0.08	+0.1918	+0.1311	+0.0887	+0.0574	+0.0268	+0.0026

Most of the pyridine atoms exhibit a systematic drop in the absolute value of the HFCC because the delocalization of the spin density via the π-space of the pyridine is less effective in the TS conformation, **Figure S2**. Atom C2 is a striking exception. Also note the inversion of the sign for atom C3 as discussed in the main text.

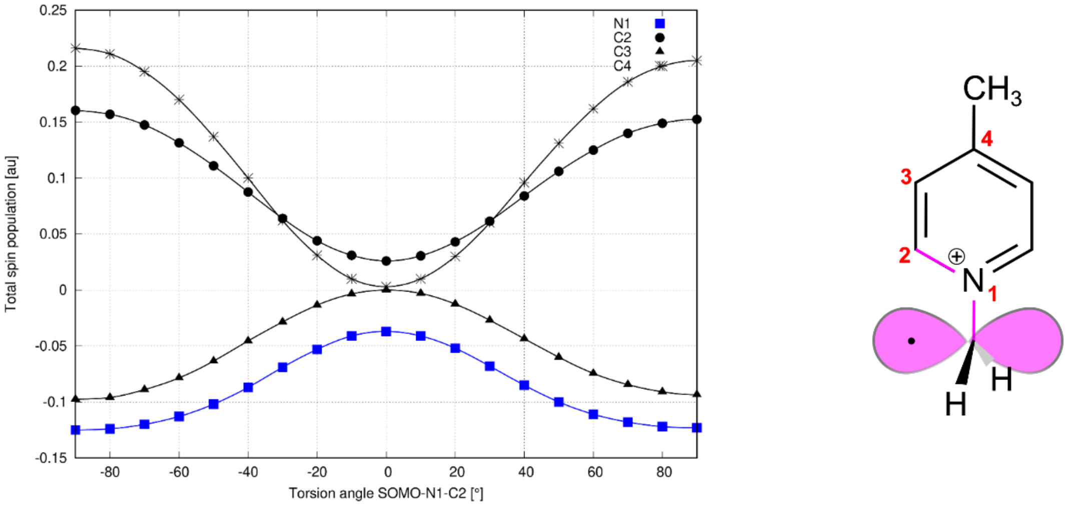

Figure S2. Total spin population for atoms N1, C2, C3, and C4 for the torsion angle SOMO-C(H_2)-N1-C2. Calculations were performed at the ZORA/PBE0/TZ2P level of theory by using Mulliken population analysis.

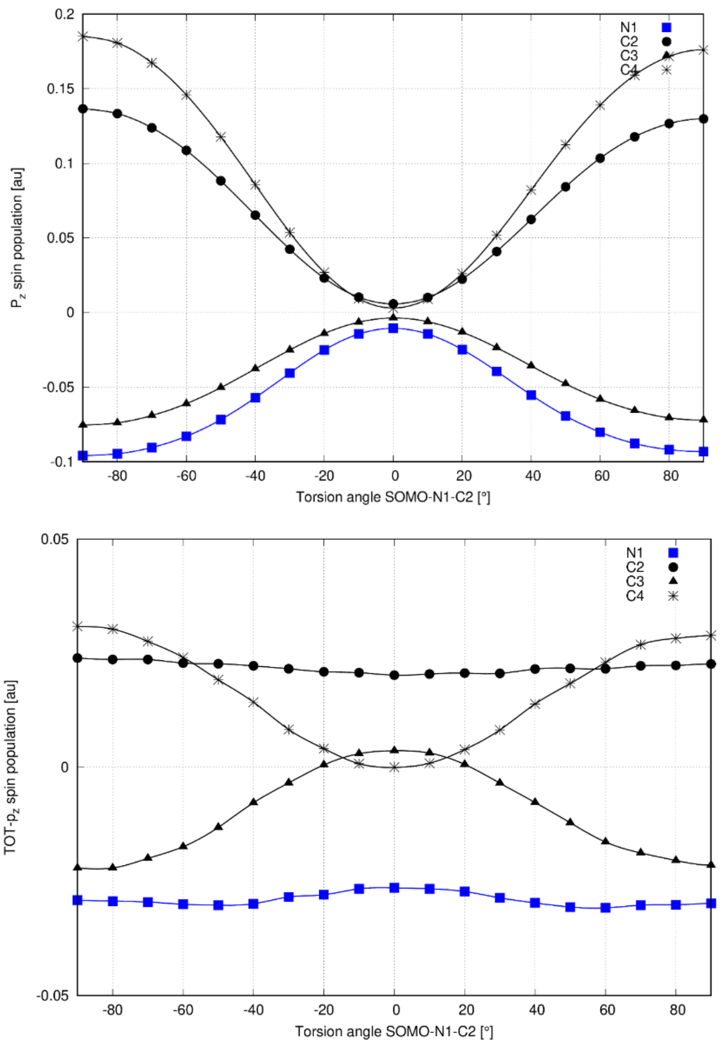

Figure S3. The p_z and total-p_z spin populations for atoms N1, C2, C3, and C4 for the torsion angle SOMO-C(H_2)-N1-C2. Calculations were performed at the ZORA/PBE0/TZ2P level of theory by using Mulliken population analysis.

Ruthenium(III) compounds

Table S2. Interatomic distance ($r_{Ru\text{-}N}$)[a], delocalization index (DI)[b], and bond energy (ΔE^{tot})[c] for the Ru–N1 bond in compounds **1-5**.

Compound (*trans*-ligand)	$r_{Ru\text{-}N}$	DI	ΔE^{tot}	ΔE^{orb}	σ-donation	π-back
**1** (F)	212	0.60	-40.8	-49.3	-29.4	-7.8
**2** (Cl)	215	0.57	-37.2	-48.5	-29.8	-6.7
**3** (CN)	224	0.48	-31.0	-35.8	-18.3	-6.8
**4** (CH_3)	234	0.38	-22.8	-28.3	-14.8	-5.2
**5** (BH_2)	256	0.26	-14.2	-16.6	-6.3	-4.6

[a] Interatomic distance Ru–N in pm.

[b] DI (Ru↔N) in a.u.

[c] EDA bond energy (Ru–N) in kcal mol^{-1}.

Table S3. Isotropic *g*-value and hyperfine coupling (HFC) constants (*A*) for individual atoms of compounds **1-5** calculated in vacuum. For structures, see **Figure 7** in the main text.

Atom	**1** (F)	**2** (Cl)	**3** (CN)	**4** (CH_3)	**5** (BH_2)
g_{iso}	2.26	2.33	2.53	2.34	2.37
**Nucleus**			A_{iso} [MHz]		
N1	-5.63	-4.63	-2.98	-2.04	+1.31
C2	+0.81	+0.14	-0.79	-0.78	-0.34
C3	-0.63	-0.72	-0.48	-0.19	+0.12
C4	+0.32	+0.31	+0.05	-0.21	-0.15
H2	-0.76	-0.80	-0.29	-0.16	0.00
H3	+0.46	+0.40	+0.24	+0.24	-0.10
HN	-1.21	-1.54	0.28	-1.96	8.79

Comment to Table S3. *Trans*-ligand effect on the HFCCs (variation of A_{iso} for individual atoms in compounds **1-5**) as transcribed to the hyperfine NMR shift can reach up to 140 ppm for ^{13}C and up to 20 ppm for ^{1}H NMR (F vs BH_2).

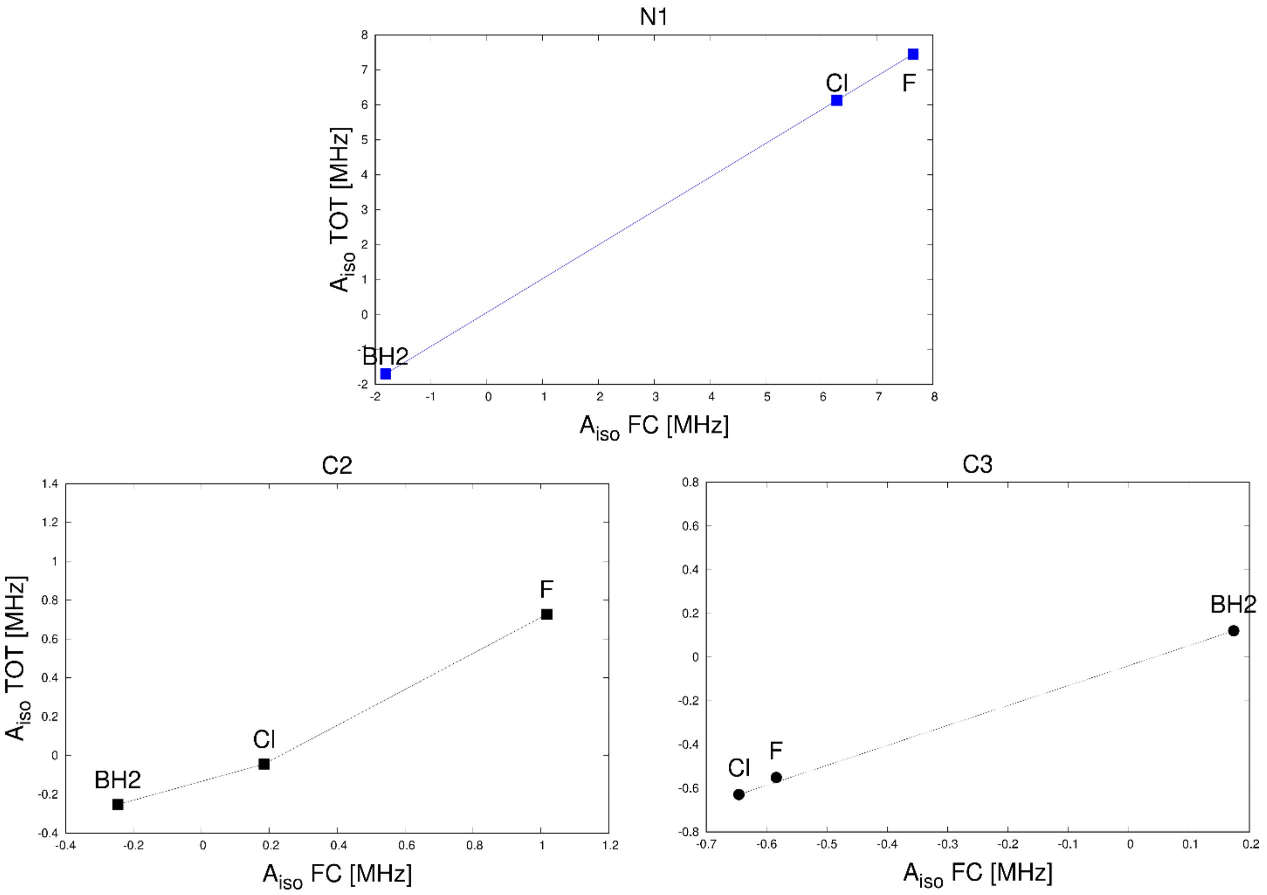

Figure S4. Correlation of the Fermi-contact contribution and total HFCC (A_{iso} in MHz) for atoms ^{14}N1, ^{13}C2, and ^{13}C3 calculated at the mDKS/PBE0/dyall-VTZ/upcJ-2 level of theory[1].

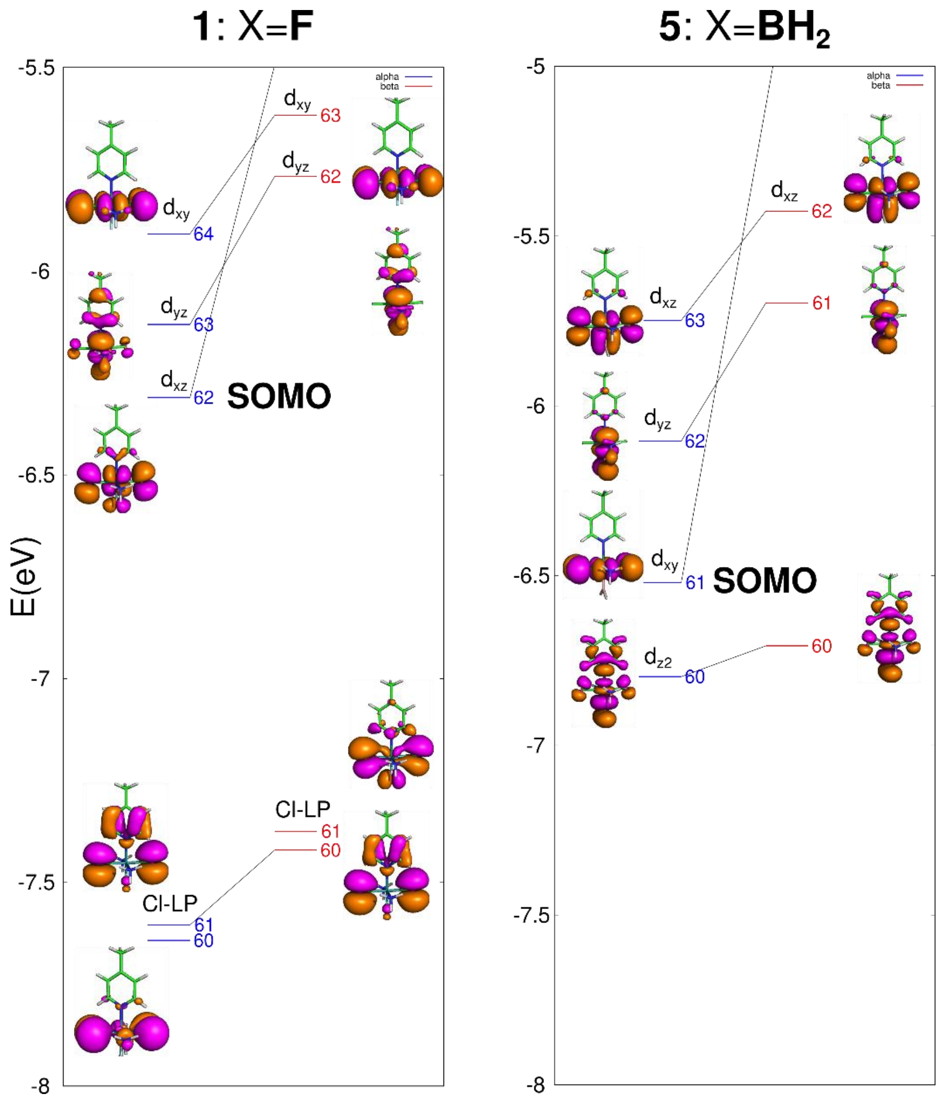

Figure S5. Energy diagram of the highest occupied Kohn-Sham MSOs (α - left in blue; β - right in red) for complexes **1** (left, X = F) and **5** (right, X = BH_2) calculated at the UKS/PBE0/def2-TZVPP level. For the ease of the reader, the α/β MSO pairs are connected by thin black lines.

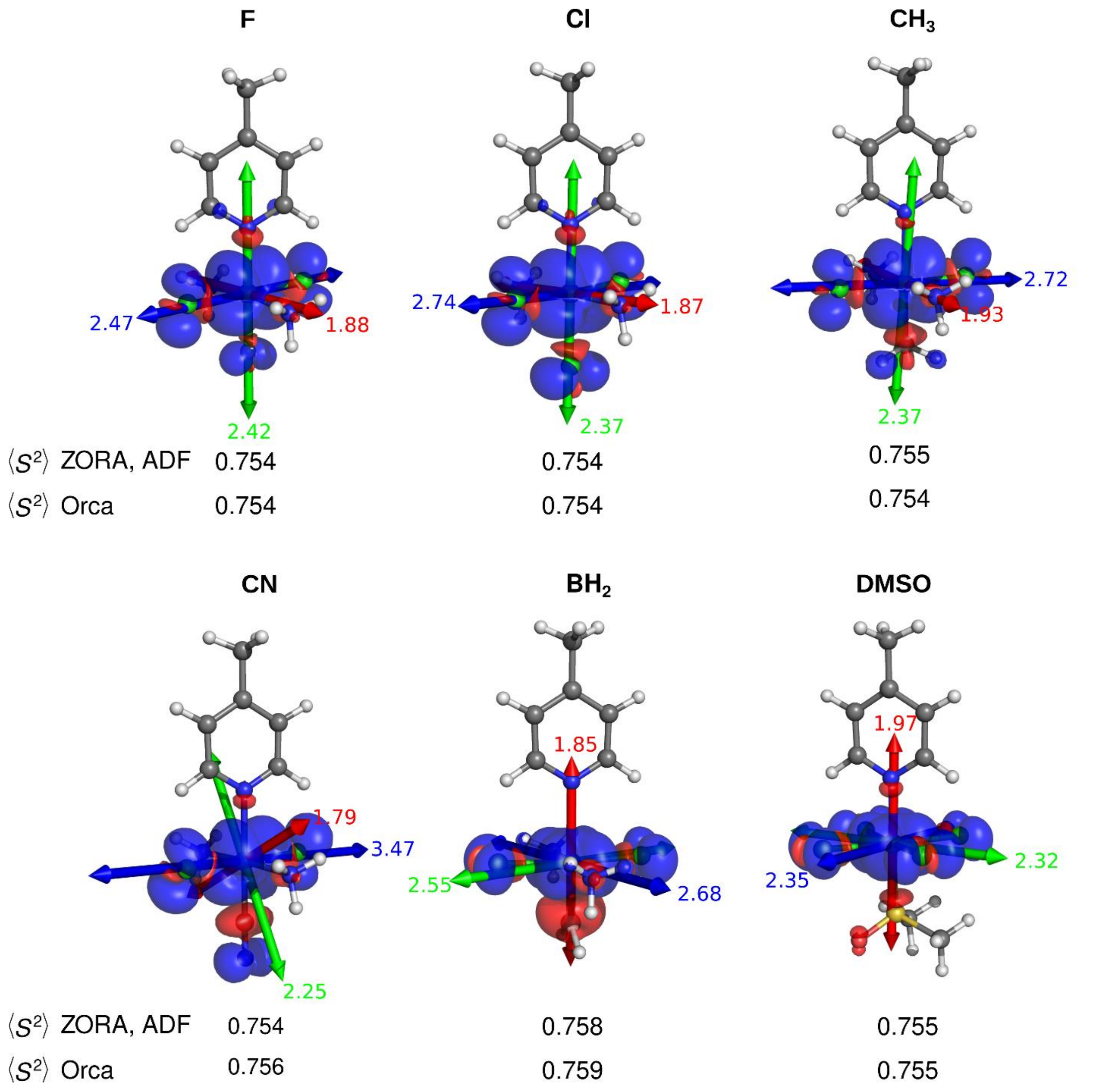

Figure S6. Visualization of the spin density (isosurface at 0.001 a.u., α in blue, β in red) in compounds **1-6** calculated at the 1c ZORA/PBE0/TZ2P level of theory. The components of the g-tensor (2c SO-ZORA/PBE0/TZ2P level) are depicted by arrows (red < green < blue).

REFERENCES

(1) Repisky, M.; Komorovsky, S.; Kadek, M.; Konecny, L.; Ekström, U.; Malkin, E.; Kaupp, M.; Ruud, K.; Malkina, O. L.; Malkin, V. G. ReSpect: Relativistic Spectroscopy DFT Program Package. *J. Chem. Phys.* **2020**, *152* (18), 184101. https://doi.org/10.1063/5.0005094.
